# Honors of Medical Men

**Published:** 1852-05

**Authors:** 


*Honors of Medical Men.*—It has pleased the Queen to grant permission to Mr. Owen, the distinguished Professor of Comparative Anatomy at the College of Surgeons, to reside in one of the houses on Kew Green which belonged to the late King of Hanover. The gift was accompanied by a very flattering letter from Prince Albert to the professor. Another of the houses on the same green has, we understand, in a like kindly spirit, been presented for a residence during life to Dr. Joseph Hooker. We have also to record a mark of royal favor bestowed by a foreign sovereign on Professor Owen. Our readers know that the King of Prussia instituted some time since an Order of Merit, for men distinguished in art and in science—and which consists of sixty chevaliers, thirty Prussians and thirty foreigners. Amongst the latter was the late Professor Oersted; and Professor Owen has been selected by Frederick William to supply the vacancy occasioned by his death. In this order there are now five Englishmen:—Mr. R. Brown, Sir David Brewster, Sir J. Herschel, Mr. Faraday, and Professor Owen.—*London Lancet.*

